# Radiomics in predicting recurrence for patients with locally advanced breast cancer using quantitative ultrasound

**DOI:** 10.18632/oncotarget.28139

**Published:** 2021-12-07

**Authors:** Archya Dasgupta, Divya Bhardwaj, Daniel DiCenzo, Kashuf Fatima, Laurentius Oscar Osapoetra, Karina Quiaoit, Murtuza Saifuddin, Stephen Brade, Maureen Trudeau, Sonal Gandhi, Andrea Eisen, Frances Wright, Nicole Look-Hong, Ali Sadeghi-Naini, Belinda Curpen, Michael C. Kolios, Lakshmanan Sannachi, Gregory J. Czarnota

**Affiliations:** ^1^Department of Radiation Oncology, Sunnybrook Health Sciences Centre, Toronto, Canada; ^2^Department of Radiation Oncology, University of Toronto, Toronto, Canada; ^3^Physical Sciences, Sunnybrook Research Institute, Toronto, Canada; ^4^Department of Medical Oncology, Department of Medicine, Sunnybrook Health Sciences Centre, Toronto, Canada; ^5^Department of Medicine, University of Toronto, Toronto, Canada; ^6^Department of Surgical Oncology, Department of Surgery, Sunnybrook Health Sciences Centre, Toronto, Canada; ^7^Department of Surgery, University of Toronto, Toronto, Canada; ^8^Department of Medical Biophysics, University of Toronto, Toronto, Canada; ^9^Department of Electrical Engineering and Computer Sciences, Lassonde School of Engineering, York University, Toronto, Canada; ^10^Department of Medical Imaging, Sunnybrook Health Sciences Centre, Toronto, Canada; ^11^Department of Medical Imaging, University of Toronto, Toronto, Canada; ^12^Department of Physics, Ryerson University, Toronto, Canada

**Keywords:** radiomics, breast cancer, quantitative ultrasound, recurrence, machine learning

## Abstract

Background: The purpose of the study was to investigate the role of pre-treatment quantitative ultrasound (QUS)-radiomics in predicting recurrence for patients with locally advanced breast cancer (LABC).

Materials and Methods: A prospective study was conducted in patients with LABC (*n* = 83). Primary tumours were scanned using a clinical ultrasound device before starting treatment. Ninety-five imaging features were extracted-spectral features, texture, and texture-derivatives. Patients were determined to have recurrence or no recurrence based on clinical outcomes. Machine learning classifiers with k-nearest neighbour (KNN) and support vector machine (SVM) were evaluated for model development using a maximum of 3 features and leave-one-out cross-validation.

Results: With a median follow up of 69 months (range 7–118 months), 28 patients had disease recurrence (local or distant). The best classification results were obtained using an SVM classifier with a sensitivity, specificity, accuracy and area under curve of 71%, 87%, 82%, and 0.76, respectively. Using the SVM model for the predicted non-recurrence and recurrence groups, the estimated 5-year recurrence-free survival was 83% and 54% (*p* = 0.003), and the predicted 5-year overall survival was 85% and 74% (*p* = 0.083), respectively.

Conclusions: A QUS-radiomics model using higher-order texture derivatives can identify patients with LABC at higher risk of disease recurrence before starting treatment.

## INTRODUCTION

Breast cancer is one of the most common cancer in women and is accountable for the leading cause of death [1]. Locally advanced breast cancer (LABC) is seen in approximately 10–30% of patients and is associated with a poor prognosis compared to early breast cancer (EBC) [2]. LABC encompasses advanced primary disease with or without metastatic involvement of regional lymph nodes. The 5-year survival for patients with LABC can vary between 50–80%, depending upon several factors including clinical characteristics and molecular features like the expression of estrogen (ER), progesterone (PR) receptors, or human epidermal growth factor receptor 2 (HER 2) expression [3–5]. Treatment typically involves a multimodality approach, including surgery, systemic therapy (chemotherapy, targeted therapy, endocrine therapy), and radiotherapy (RT). Translational and clinical research are investigating different strategies to improve outcomes and developing up-front biomarkers to identify patients at higher risk of disease recurrence. Several genetic tests are available in predicting tumour aggressiveness in parallel to what is classically ascertained through tissue assessment, with their role well established for early breast cancer [6–9]. Such tests have found utility in guiding clinicians to decide the role of treatment intensification (particularly chemotherapy) in patients predicted to harbour a relatively higher risk of disease. However, the application of such biomarkers is limited in LABC, where clinical outcomes could be potentially improved given the higher risk of relapse.

Imaging in oncology has a well-established role in diagnosis, staging, response assessment, and surveillance. In recent years, there has been a paradigm shift with the introduction of artificial intelligence in medicine, with the promising role of imaging to be used as a noninvasive biomarker in understanding tumour biology [10, 11]. More popularly known as “radiomics,” advanced imaging analysis has generated promise in disease stratification and predicting clinical outcomes. Several imaging modalities like mammography (MMG), ultrasonography (USG), computed tomography (CT), magnetic resonance imaging (MRI), and positron emission tomography (PET) have proved utility in the field of radiomics for breast cancer [12, 13]. Quantitative ultrasound (QUS) is similar to conventional USG with the advantage of capturing and analysis of raw radiofrequency (RF) data, which can better characterize tissue microstructure [14, 15]. The basis for QUS is its ability to detect the microstructural elastic properties, which are different between benign or malignant tissues or also between various grades of tumours. The commonly used spectral features include mid-band fit (MBF), spectral slope (SS), spectral intercept (SI), spacing among scatterers (SAS), acoustic scatterer diameter (ASD), average acoustic-scatterer concentration (AAC), and attenuation coefficient estimate (ACE). Texture analysis from spectral images using gray level co-occurrence matrix (GLCM) can extract second-order imaging features like contrast (CON), correlation (COR), energy (ENE), and homogeneity (HOM), which can provide insights into different aspects of tumour heterogeneity. Studies have demonstrated the clinical efficacy of QUS in predicting response to neoadjuvant chemotherapy (NAC) in LABC [16–20], and in patients with head-neck malignancies treated with radiotherapy [21].

In this study, we investigated the role of QUS obtained before the start of treatment in predicting the risk of tumour recurrence in patients with LABC. The imaging features were obtained from the QUS imaging, which included spectral parameters, texture of spectral parameters (QUS-Tex^1^), and second-order texture analysis of QUS-Tex^1^ features (QUS-Tex^1^-Tex^2^). Model development was done using *k-*nearest neighbours (KNN) and support vector machines-radial basis function (SVM). To the best of our knowledge, this is the first study of QUS-radiomics to predict the recurrence groups in patients with LABC.

## RESULTS

### Clinical features

A total of 83 patients were included in the final analysis. The median follow up was 69 months (range 7–118 months) for all patients and 74 months (range 49–118 months) for patients without any evidence of disease recurrence. The total number of patients with recurrence was 28, whereas 55 were free from any recurrence until the last follow up. The distribution of various features between the two patient groups (recurrence versus non-recurrence) is summarized in Table 1. The most common histological type was invasive ductal carcinoma, found in 92% of patients. The majority of the patients had hormone-positive disease with ER+ and PR+ status in 58% and 52% of patients, respectively. Her2 expression was observed in 35% of patients.

**Table 1:** Clinical characteristics for the two groups (recurrence vs. no recurrence)

Features		Recurrence (*n* = 28)		No recurrence (*n* = 55)	
Variables	Categories	*n*	%	*n*	%
**Age**	Median (Range)	50 (29–79) years		48 (31–72) years	
**Menstrual status**	Premenopausal	16	56	33	60
	Perimenopausal	1	4	3	6
	Postmenopausal	10	36	17	30
	Unknown	1	4	2	4
**Laterality**	Right	15	54	27	49
	Left	13	46	28	51
**Histology**	IDC	25	89	51	92
	ILC	2	7	1	2
	Others	1	4	3	6
**ER Status**	Negative	13	46	22	40
	Positive	15	54	33	60
**PR Status**	Negative	13	46	27	49
	Positive	15	54	28	51
**HER2 Status**	Negative	18	64	36	66
	Positive	10	36	19	34
**T stage**	T1	0	0	0	0
	T2	7	25	28	50
	T3	13	46	24	44
	T4	8	29	3	6
**N stage**	N0	5	18	15	27
	N1	16	57	34	61
	N2	4	14	3	6
	N3	3	11	3	6

Abbreviations: IDC: Invasive ductal carcinoma; ILC: Invasive lobular carcinoma; ER: Estrogen receptor; PR: Progesterone receptor; HER2: Human epidermal receptor 2.

### Survival outcomes

The 3 and 5-year recurrence-free survivals (RFS) for the entire cohort were 77% and 68%, respectively. The median time to recurrence was 24 months (range 4–82 months). Out of all recurrences, more than 80% occurred in the initial 4 years. The predominant pattern of initial recurrence was distant metastasis (DM) in 22 patients, followed by local relapse in 7, and regional nodes in 6 (Supplementary Figure 1). The common sites of DM were bone, lung, and liver in 61%, 54%, and 46% of patients, respectively. The 3 and 5-year overall survival (OS) in the entire group was 89% and 79%, respectively.

### Feature analysis and classifier performances

**Figure 1:**
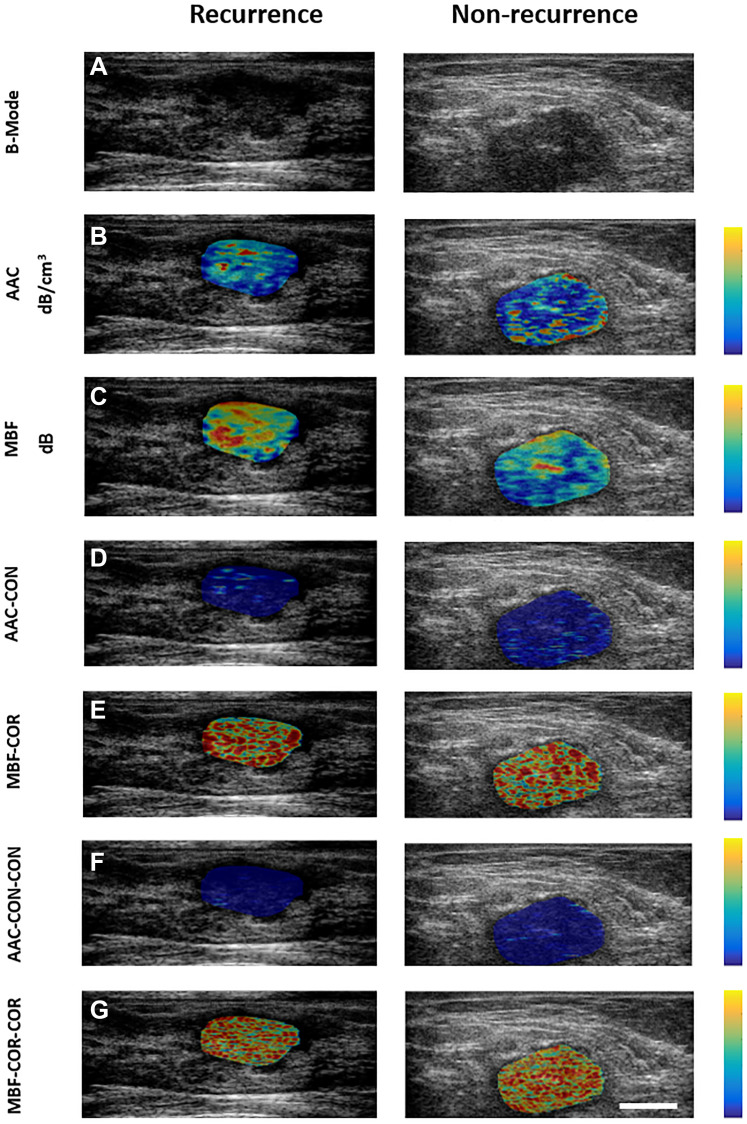
(**A**) Pre-treatment B-Mode images, QUS parametric maps ((**B**) AAC, (**C**) MBF), QUS-texture maps ((**D**) AAC-CON, (**E**) MBF-COR), and QUS-texture derivative maps ((**F**) AAC-CON-CON, (**G**) MBF-CR-COR) for one patient with recurrent disease (left panel) and one without recurrence (right panel). The colour-coded maps are generated over the region corresponding to the tumour using normalized values for individual features within the sub-ROIs. The colour scale on the right side represents the values for the individual features (B) 7 to 64 dB/cm^3^, (C) −21 to 21 dB, (D) 0 to 34, (E) −0.43 to 0.94, (F) 0 to 53, and (G) −0.54 to 0.94. The scale bar represents 2 cm.

**Figure 2:**
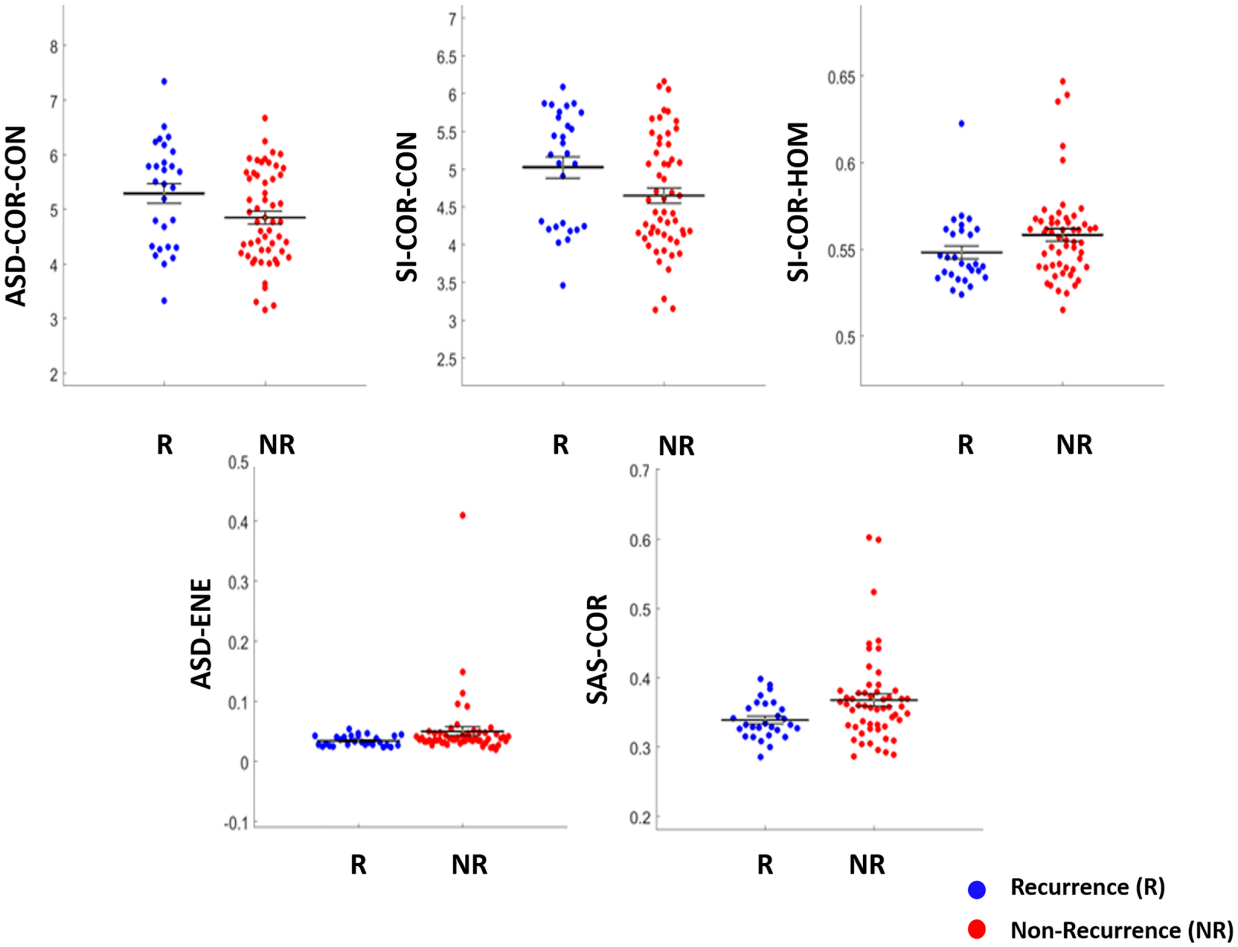
Scatter plots showing the features with the difference in distribution between the two groups (Recurrence vs. Non-recurrence) reaching the threshold of statistical significance (*p* < 0.05).

The representative B-mode, QUS parameter, texture and texture-derivative parametric maps for one patient, each with and without any recurrence, are presented in Figure 1. Two QUS-Tex^1^ features had a significantly different distribution between the two groups- SAS-COR (*p* = 0.025), ASD-ENE (*p* = 0.026) (Table 2). Another three QUS-Tex^1^-Tex^2^ features exhibited significant differences-ASD-COR-CON (*p* = 0.042), SI-COR-CON (*p* = 0.033), and SI-COR-HOM (*p* = 0.049). The scatter plots indicating the distributions of these features between the two groups are presented in Figure 2. The scatter plots for all the 95 features have been included in Supplementary Figure 2.

**Table 2:** Features with differential distribution between the two groups with statistical significance

Parameter	Recurrence	No recurrence	*p*-value
	Mean ± SEM	Mean ± SEM	
SAS-COR	.3396 ± .02734	.3684 ± .06400	0.025
ASD-ENE	.0354 ± .00853	.0510 ± .05399	0.026
ASD-COR-CON	5.30 ± 0.95	4.85 ± 0.85	0.042
SI-COR-CON	5.0289 ± .75007	4.6532 ± .76247	0.033
SI-COR-HOM	.5484 ± .01998	.5585 ± .02659	0.049

Abbreviations: SEM: Standard error of the mean; SAS: Spacing among scatterers; ASD: Acoustic scatterer diameter; SI: Spectral slope; COR: Correlation; ENE: Energy; CON: Contrast; HOM: Homogeneity.

**Figure 3:**
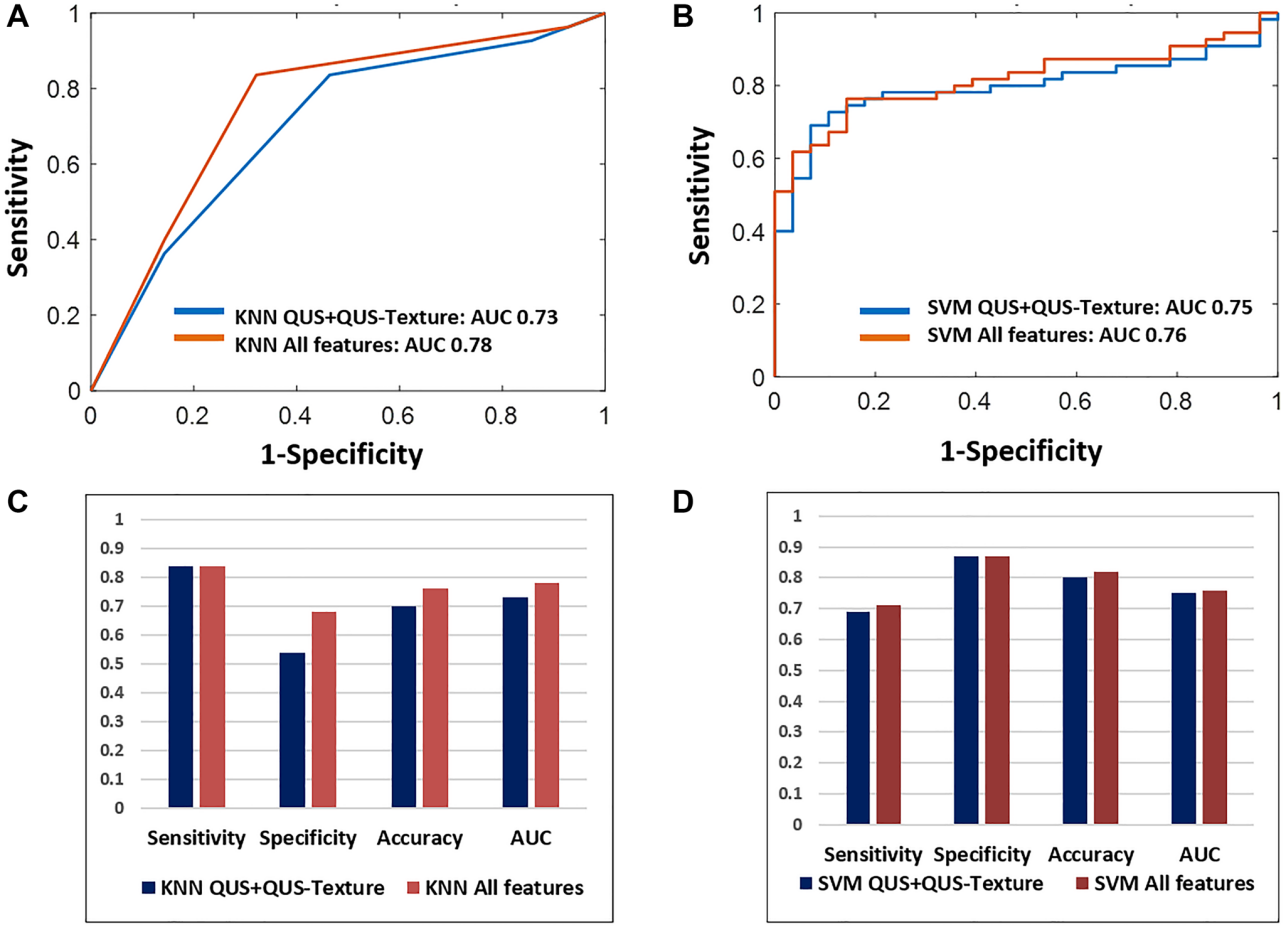
The classifier indices for the two machine learning classifiers. (**A** and **B**) show the ROC curves using KNN and SBM classifiers, respectively, showing the effect of inclusion of higher-order imaging features (texture-derivatives). (**C** and **D**) are the bar diagrams representing the diagnostic indices (sensitivity, specificity, accuracy, and AUC) for the KNN and SVM model with and without the use of texture-derivatives.

The classifier performances using KNN and SVM are summarized in Table 3. The best result was obtained with the SVM classifier (selecting from all 95 features) with a sensitivity, specificity, accuracy, and AUC of 71%, 87%, 82%, and 0.76, respectively. Using the KNN model and again selecting from all features, the sensitivity, specificity, accuracy, and area under curve (AUC) were 84%, 68%, 76%, 0.78, respectively. The inclusion of third-order features (QUS-Tex^1^-Tex^2^) further improved the diagnostic performances remarkably for the KNN classifier from 70% to 76%, while for SVM, it had only a small performance increment, changing from 80% to 82%. The corresponding ROC plots for the models are presented in Figure 3A and 3B and the representative bar diagram in Figure 3C and 3D.

**Table 3:** Classification performance of the two machine learning classifiers with the best-selected features

Classifier	Features	Sensitivity (%)	Specificity (%)	Accuracy (%)	AUC	Selected features
**KNN**	**QUS+ QUS-Tex^1^**	84	54	70	0.73	ASD-COR SAS-HOM SAS-ENE
	**QUS+ QUS-Tex^1^+** **QUS-Tex^1^-Tex^2^**	84	68	76	0.78	ACE AAC-CON-CON AAC-ENE-HOM
**SVM**	**QUS+ QUS-Tex^1^**	69	87	80	0.75	SAS ASD-CON MBF-COR
	**QUS+ QUS-Tex^1^+** **QUS-Tex^1^-Tex^2^**	71	87	82	0.76	SAS ASD-CON MBF-COR

Abbreviations: KNN: k-nearest neighbour; SVM: Support vector machine; AUC: Area under curve; QUS: Quantitative ultrasound; QUS-Tex^1^: QUS-texture; QUS-Tex^1^-Tex^2^: QUS-texture derivatives.

**Figure 4:**
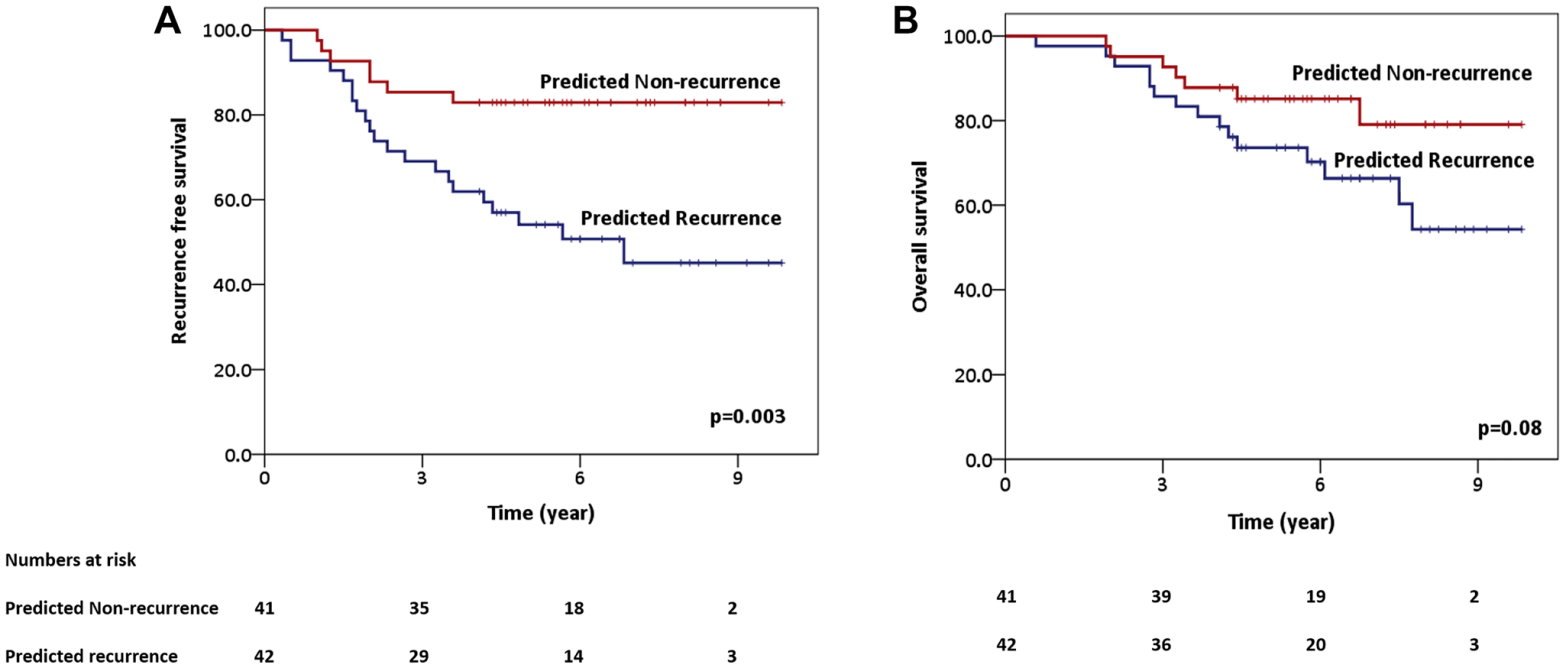
Predicted survival plots using support vector machine classifier predicted groups (predicted recurrence vs. predicted non-recurrence)-recurrence-free survival (**A**) and overall survival (OS) (**B**).

Finally, the model-based predicted groups were evaluated to investigate the impact on RFS and OS. The SVM model performed best in segregating the two groups, with the predicted 5-year RFS being 83% (predicted non-recurrence) versus 54% (predicted recurrence), with a *p*-value of 0.003. The 5-year OS for the predicted non-recurrence and recurrence groups using the SVM classifier was 85% and 74%, respectively (*p* = 0.08). The estimated RFS and OS plots using the SVM model have been shown in Figure 4.

## DISCUSSION

Breast cancer represents a heterogeneous disease entity with survival dependant upon several clinical, biological, and treatment-related factors. *In-situ* and early breast cancer lie on one end of the spectrum with excellent survival rates, whereas metastatic breast cancer represents a disease with a dismal prognosis [22–24]. Locally advanced breast cancer is associated with an intermediate prognosis, with disease recurrence more commonly encountered than earlier breast cancer. In recent decades, the identification of specific molecular pathways and the availability of systemic and targetted agents have helped to improve the outcomes to a certain extent. Still, there is an unmet need to develop biomarkers, which can further help refine existing risk-stratification and pave the way towards precision and personalized medicine. The study presented here presents a novel strategy of using imaging like QUS and artificial intelligence-based tools to predict patients with a higher risk of recurrence before initiating any treatments.

Several genetic markers have established their role in stratifying risk recurrence in patients with EBC [6–8]. A 21-gene recurrence score had been used to guide treatment, particularly in EBC, showing improvement of clinical outcomes with consideration of adjuvant systemic therapy and treatment de-escalation in low-risk patients [25, 26]. However, there is limited literature related to such genetic markers with clinical application in patients with LABC. Molecular profile has been shown to influence the recurrence risk in LABC, with ER/PR+/HER2- tumours having better outcomes as compared to triple-negative cancers [27, 28]. Circulating microRNA and DNA methylation have been indicated to correlate with the clinical outcomes in patients with LABC [29, 30]. The introduction of radiomics has shown promise of developing noninvasive biomarkers in risk stratification and response monitoring. Several radiomic studies have been adopted in breast cancer patients using different imaging modalities [12, 13]. Radiomic analysis of MRI scans has been correlated with the established genetic tools in prognostication of patients with breast cancer [31]. In a study including 294 patients with breast cancer, Park et al. demonstrated an MRI-based radiomic signature correlated with disease-free survival [32]. QUS-Radiomics obtained before starting treatment and early during NAC have also predicted patients’ final pathological response with LABC [16, 17]. Ha et al. demonstrated the utility of radiomic features obtained from PET/CT to be associated with treatment response and prognosis in patients with LABC [33].

Ultrasound is a widely available portable imaging modality with rapid scan acquisition and significantly lower costs than other imaging modalities. Conventional B-mode US is commonly used in screening and diagnostic evaluation [34, 35], with morphological features demonstrating correlation with breast cancer biology to some extent [36, 37]. Compared to conventional B-mode imaging, QUS provides more detailed information as obtained from the unprocessed radiofrequency data and is machine-independent, thus less influenced by technical variations or subjective interpretations. QUS spectral parameters have been demonstrated to be related to tissue microstructural elastic properties. In preclinical studies, QUS has been shown to effectively detect cell death associated with various treatment modalities [14, 38]. Further clinical studies have demonstrated the efficacy of pre-chemotherapy QUS-radiomics in predicting responses to NAC with an accuracy of 88% [16]. In the current study, the pre-treatment QUS-radiomics parameters could be used to identify patients developing disease recurrence with an accuracy of 82% using an SVM-based model. The SAS (scatterer spacing) parameter was one of the selected features in the SVM model, suggesting a differential microstructural organization and architecture between the two risk groups with its influence on biological behaviour. In a previous study, the SAS and associated textural features were shown to help differentiate grade I from grade II/ III breast cancer, with tumour grade known to reflect cancer differentiation and aggressiveness [39]. The other two features selected in the SVM model were ASD-CON and MBF-COR (texture features). The ASD parameter reflects the microstructural size, while MBF represents scatterer size, shape, number, organization, and elastic properties. The texture features are particularly helpful in characterizing tissue heterogeneity. Intratumoral heterogeneity is a well-known entity in breast cancer, leading to the evolution of therapeutic resistance and the development of disease recurrence [40, 41]. It is, therefore, reasonable to assume during presentation that high-risk and low-risk tumours harbour diverse levels of intratumoral heterogeneity, which can be detected by advanced imaging analysis methods. QUS probes into tumour structure and is influenced by the organization at the cellular level. In this regard, it is important to note that third-order imaging features or texture-derivatives significantly improved here of the KNN model from 70% to 76% (modest improvement for SVM). It is possible the higher-order imaging features can further detect the heterogeneity at a deeper level helping in the refinement of the radiomics model.

The 5-year RFS of 68% and 5-year OS of 79% in our study is comparable to previous reports [3–5]. As noted earlier, in EBC, genetic-based recurrence scores had initially shown the way towards the development of risk-adapted treatment protocols [26, 42]. In the neoadjuvant setting, response-guided chemotherapy [43] or the addition of bevacizumab to docetaxel and trastuzumab [44] has been demonstrated to improve disease-free survival and response rates. It is crucial to identify in advance patients with an aggressive disease having a higher risk of recurrence, which can help prognosticate and design appropriate treatment intensification strategies. For instance, high-risk LABC patients could be investigated for additional systemic treatment options, including maintenance therapies, given the higher risk of distant metastases. Similarly, some patients clinically presenting with LABC can have biologically relatively indolent tumours, and careful de-escalation strategies can be explored in such cohorts avoiding or minimizing treatment-related toxicities.

### Limitations and future directions

The study presented here has a relatively small number of patients and has been expanded to continue in a larger population. It is possible with a higher number of patients, advanced strategies like deep learning can be used to improve classification performance and the reliability of generated radiomics models. Although we had a relatively longer follow up (median follow up >6 years in patients without recurrence), small groups can exhibit late recurrence with a possible switch of the output groups. Based on the use of QUS-radiomics to predict the response to NAC, a randomized trial is currently underway to study the effect of adaptive chemotherapy (https://clinicaltrials.gov identifier NCT04050228).

## MATERIALS AND METHODS

### Patient selection and treatment

This prospective, observational study was approved by the Sunnybrook Health Sciences Centre Research Ethics Committee and registered (https://clinicaltrials.gov identifier NCT00437879). The research was conducted following good clinical practice and as per the declaration of Helsinki. Written consent was obtained for all study participants. Patients with a diagnosis of LABC were deemed eligible for the study. All the patients were treated with neoadjuvant chemotherapy (NAC) followed by surgery in the form of breast-conserving surgery or mastectomy with sentinel lymph node biopsy or axillary dissection (according to clinical standards and patient preferences). Additional subsequent adjuvant treatment was carried out with radiation and systemic therapies (endocrine therapy, targeted therapy) according to standard institutional practice. Patients were seen in the clinic every 3–6 months in the first 2 years, and after that every 6–12 months or as indicated clinically. Recurrence was confirmed by clinical investigations, medical imaging and supplemented with tissue diagnosis where available and appropriate, based on the decision of the treating physicians. Patients without any evidence of recurrent disease who had a minimum follow up of 4 years or recurrence (any time point within 4 years) were included in this analysis. Patients with recurrence suggesting a new breast primary (e.g. recurrence involving different quadrant or contralateral breast) or second different primary malignancy (e.g. lung primary) were excluded from the analysis, as they were likely to be associated with different biological etiology.

### Acquisition of ultrasound data

All patients were scanned using a clinical ultrasound device Sonix RP (Analogic Medical Corp., Vancouver), with a linear array transducer having a central frequency of 7 MHz (bandwidth 4–9 MHz). For digital RF data acquisition, a sampling frequency of 40 MHz was used with a 16-bit resolution. Scans were obtained before the initiation of any cancer-directed treatment. For imaging, the transducer was focused towards the centre of the tumour and was scanned at regular intervals of 1 cm. The primary tumour was contoured manually and designated as the region of interest (ROI).

### Image analysis

RF Data corresponding to the ROI was analyzed using a sliding window analysis with an overlap of 94% in the axial and lateral directions to generate sub-regions of interest (sub-ROI). The corresponding dimensions of the sub-ROIs were 2 mm x 2 mm. The RF data obtained from each sub-ROI was subjected to a fast Fourier transform (FFT) and normalized using a tissue-mimicking phantom to obtain power spectra. Individual QUS parameters were determined throughout the entire tumour-MBF, SS, SI, SAS, ASD, and AAC. An ACE parameter was used in the spectral correction and served as an independent feature as well. The QUS parameters from all the sub-ROIs were determined, and the final values from the entire ROI served as first-order imaging features. Further details related to data processing are described in previous publications [39].

For the determination of second-order features, first, a parametric-coded map was generated for each of the QUS parameters (except ACE), accounting for individual values within the sub-ROIs, called QUS parametric maps. Texture analysis was carried out subsequently using GLCM, which computes the relation of the index pixel with the neighbouring ones at four different angular relations, 0°, 45° 90°, and 135°. Four GLCM features were analyzed-CON, COR, ENE, and HOM. These led to a total of 24 QUS-Tex^1^ features (from 6 QUS parametric maps, no parametric map generated for ACE).

In order to obtain the third-order imaging parameters, 16 QUS-Tex^1^ features were used (the texture maps of SS and SAS were not used). Firstly, a colour-coded map was regenerated from the values obtained from the texture values corresponding to the sub-ROIs. In a similar manner, the 4 GLCM texture analysis was repeated, which led to 64 QUS-Tex^1^-Tex^2^ features. The study proceeded with a total of 95 features (7 QUS, 24 QUS-Tex^1^, 64 QUS-Tex^1^-Tex^2^). Each of these features was averaged from all the tumour slices obtained from a single patient, and the mean value was used for final analysis.

### Statistical analysis and machine learning classifiers

The patients were labelled using binary classes depending upon the final clinical outcomes, including recurrence (R) and non-recurrence (NR). The distribution of each feature between the two groups was tested using a Shapiro-Wilk test. Unpaired *t*-tests and Mann-Whitney tests were conducted for normally distributed data and non-parametric data, respectively. A *p*-value of <0.05 was considered statistically significant. Two machine learning classifiers were used for model development using forward feature selection algorithms- KNN and SVM. A maximum of three features was used for classification to avoid overfitting of data into the model. As the number of individuals was unevenly distributed between the two groups (recurrence versus non-recurrence), the data was balanced divided into several subsets prior to the application of the machine learning classifiers. Subsets were selected randomly from the entire group selecting proportionately equal number of patients with and without disease recurrence. Leave-one-out cross-validation was used to test the reliability and obtain the confusion matrix for the classifiers. The data were analyzed independently using first-order and second-order features (QUS+ QUS-Tex^1^) and then incorporating both using all 95 features (QUS+ QUS-Tex^1^+ QUS-Tex^1^-Tex^2^) to evaluate the impact of higher-order features on classification performances. The AUC was obtained from receiver operating characteristics (ROC) analyses. Also, other indices like sensitivity, specificity, accuracy were obtained and compared between the outputs from the different classifiers. For processes of tumour segmentation, data processing, data extraction, and machine learning classification MATLAB R2011B (Mathworks, USA) were used. The Kaplan Meier product-limit method was used for survival analysis using the Statistical Package for the Social Sciences (SPSS V21, IBM Corporation, Armonk, New York, USA). The date of starting NAC was considered as the baseline for survival analysis. The final performance of the classifier models was tested using log-rank tests (comparison of survival rates between predicted recurrence and predicted non-recurrence groups).

### Ethics approval and consent

The study was conducted following the Declaration of Helsinki. The ethics committee of Sunnybrook Health Sciences Centre, Toronto, was involved in study approval, necessary data monitoring, and appropriate conduct of the research. The participants signed a written consent form prior to study accrual.

### Data availability

Anonymized data will be shared based on an individual request in compliance with the institutional ethics committee policies.

## CONCLUSIONS

QUS-Radiomic features obtained before the start of treatment can predict the risk of disease recurrence with reasonable accuracy. The incorporation of higher-order imaging features in the form of texture derivatives leads to the improvement of the classifier performances. The noninvasive imaging biomarker can lead to future strategies in the prognostication of patients with LABC and pave the way towards personalized medicine.

## SUPPLEMENTARY MATERIALS



## Abbreviations

LABC: Locally advanced breast cancer
EBC: Early breast cancer
ER: Estrogen receptor
PR: Progesterone receptor
HER 2: Human epidermal growth factor receptor 2
RT: Radiotherapy
MMG: Mammography
USG: Ultrasonography
CT: Computed tomography
MRI: Magnetic resonance imaging
PET: Positron emission tomography
QUS: Quantitative ultrasound
RF: Radiofrequency
MBF: Mid-band fit
SS: Spectral slope
SI: Spectral intercept
SAS: Spacing among scatterers
ASD: Acoustic scatterer diameter
AAC: Average acoustic-scatterer concentration
ACE: Attenuation coefficient estimate
GLCM: Gray level co-occurrence matrix
CON: Contrast
COR: Correlation
ENE: Energy
HOM: Homogeneity
NAC: Neoadjuvant chemotherapy
QUS-Tex^1^: QUS-texture
QUS-Tex^1^-Tex^2^: QUS-texture derivatives
KNN: *k*-nearest neighbor
SVM: Support vector machine
RFS: Recurrence-free survival
DM: Distant metastasis
OS: Overall survival
ROI: Region of interest
NR: Non-recurrence
R: Recurrence
