# Supplementary material for: Radiomics in predicting recurrence for patients with locally advanced breast cancer using quantitative ultrasound
Source: Oncotarget. 2021 Dec 7;12(25):2437–48. doi: 10.18632/oncotarget.28139 (PMC8664392; doi:10.18632/oncotarget.28139)
Supplement: Supplementary file 1 [file oncotarget-12-2437-s001.pdf]

Radiomics in predicting recurrence for patients with locally advanced breast cancer using quantitative ultrasound

SUPPLEMENTARY MATERIALS

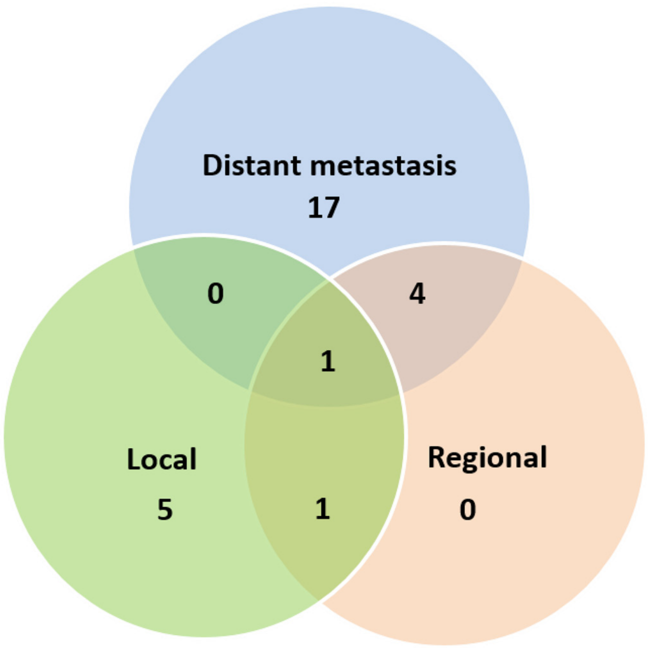

Supplementary Figure 1: Venn diagram representing the pattern of relapse (local, regional or distant) when first diagnosed with disease recurrence.

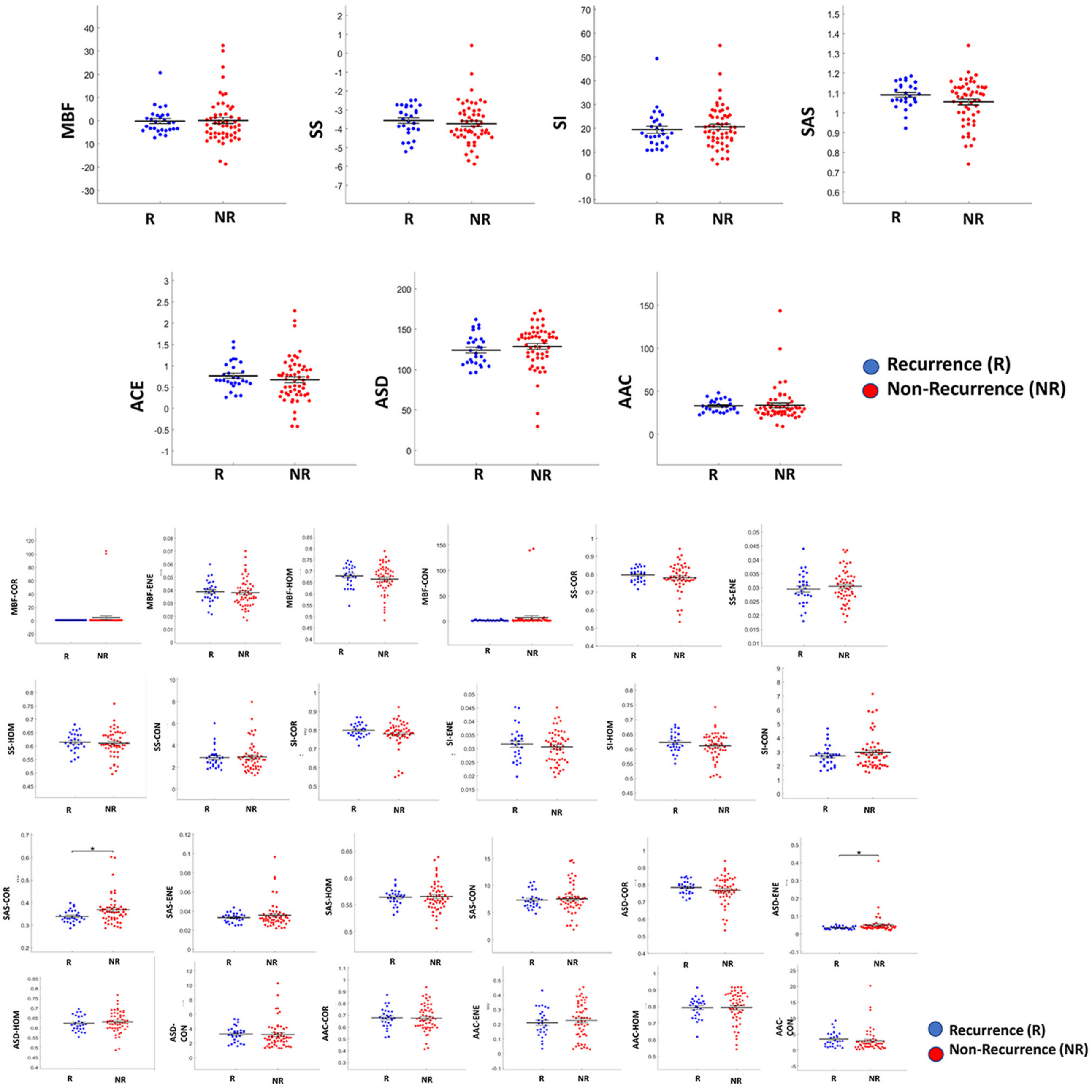

MBF
SS
SI
SAS
ACE
ASD
AAC
R
NR
Recurrence (R)
Non-Recurrence (NR)
MBF-COR
MBF-ENE
MBF-HOM
MBF-CON
SS-COR
SS-ENE
SS-HOM
SS-CON
SI-COR
SI-ENE
SI-HOM
SI-CON
SAS-COR
SAS-ENE
SAS-HOM
SAS-CON
ASD-COR
ASD-ENE
ASD-HOM
ASD-CON
AAC-COR
AAC-ENE
AAC-HOM
AAC-CON
*

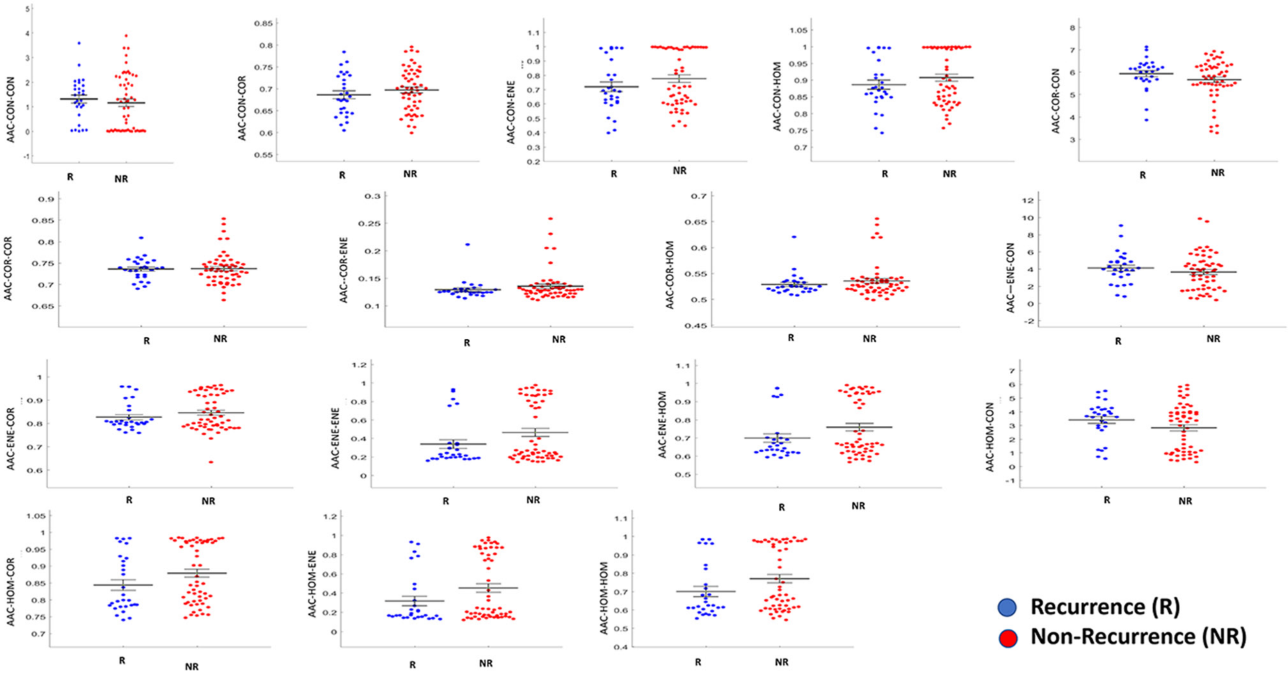
AAC-CON-CON
AAC-CON-COR
AAC-CON-ENE
AAC-CON-HOM
AAC-COR-CON
AAC-COR-COR
AAC--COR-ENE
AAC-COR-HOM
AAC—ENE-CON
AAC-ENE-COR
AAC-ENE-ENE
AAC-ENE-HOM
AAC-HOM-CON
AAC-HOM-COR
AAC-HOM-ENE
AAC-HOM-HOM
R
NR
Recurrence (R)
Non-Recurrence (NR)

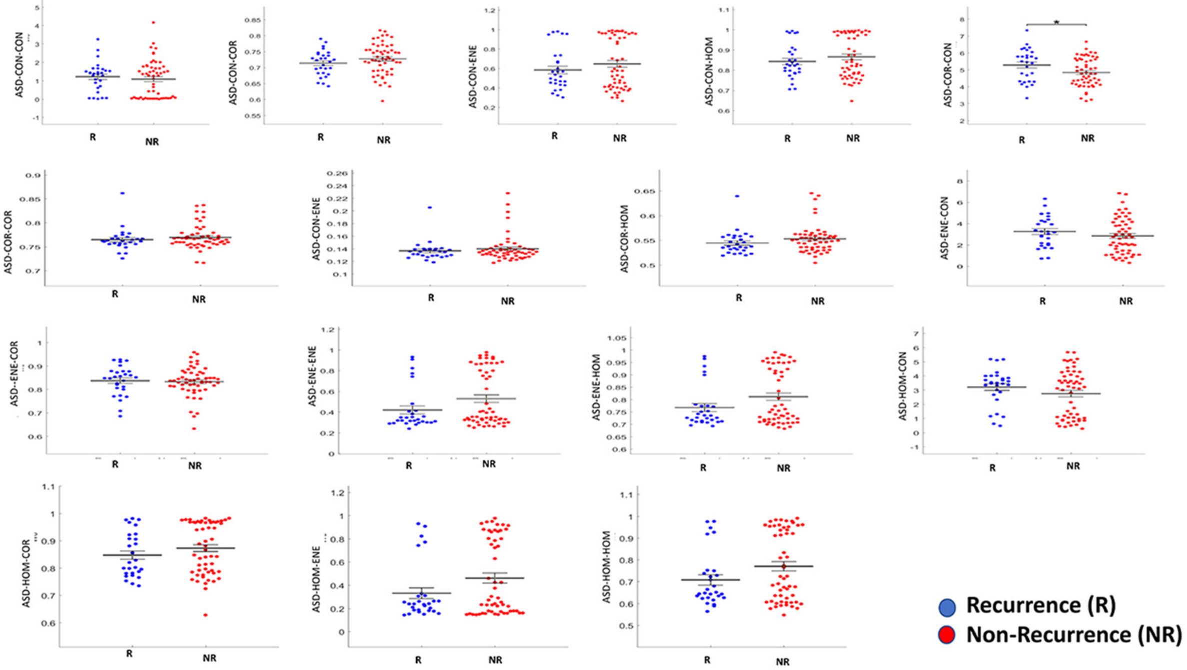
ASD-CON-CON
ASD-CON-COR
ASD-CON-ENE
ASD-CON-HOM
ASD-COR-CON
*
ASD-COR-COR
ASD-CON-ENE
ASD-COR-HOM
ASD-ENE-CON
ASD--ENE-COR
ASD-ENE-ENE
ASD-ENE-HOM
ASD-HOM-CON
ASD-HOM-COR
ASD-HOM-ENE
ASD-HOM-HOM
R
NR
Recurrence (R)
Non-Recurrence (NR)

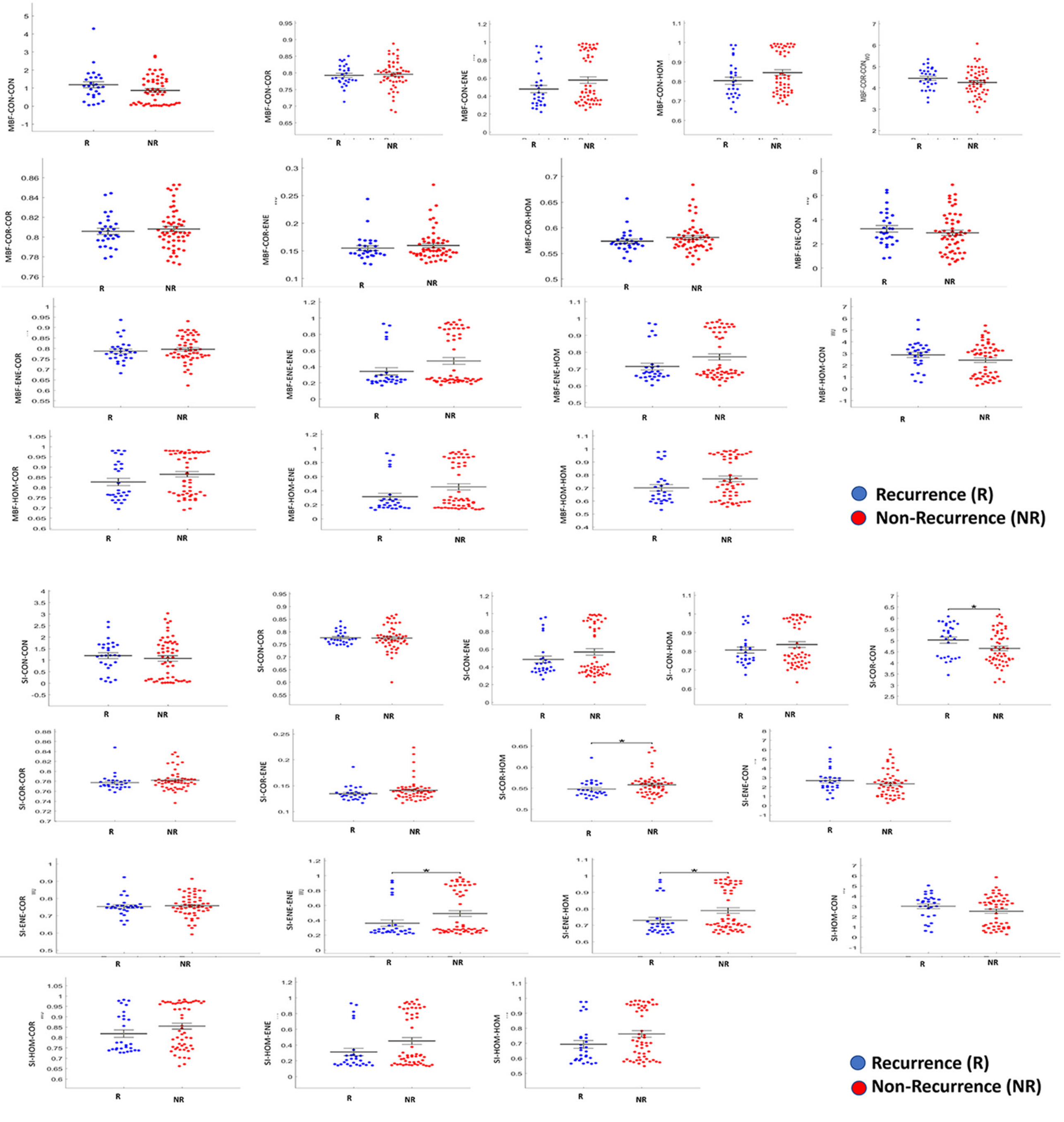

Supplementary Figure 2: Scatter plots of all 95 features showing the distribution between the two groups (Recurrence vs. Non-recurrence).
